# Comparison of Biological Agent Monotherapy and Associations Including Disease-Modifying Antirheumatic Drugs for Rheumatoid Arthritis: Literature Review and Meta-Analysis of Randomized Trials

**DOI:** 10.3390/jcm12010286

**Published:** 2022-12-29

**Authors:** Célia Delpech, François-Xavier Laborne, Pascal Hilliquin

**Affiliations:** 1 Department of Rheumatology, Centre Hospitalier du Sud Francilien, 91100 Corbeil-Essonnes, France; 2 Clinical Research Unit, Centre Hospitalier du Sud Francilien, 91100 Corbeil-Essonnes, France

**Keywords:** rheumatoid arthritis, DMARDs (synthetic), biologic agents, systematic reviews, meta-analysis

## Abstract

Objective: Update the available evidence comparing biologic disease-modifying antirheumatic drugs (bDMARDs) in combination with conventional synthetic disease-modifying antirheumatic drugs (CsDMARDs) to bDMARDs in monotherapy in patients with rheumatoid arthritis. Methods: Research was limited to randomized controlled trials. Major outcome: ACR 20 response criteria at 24 weeks. Secondary outcomes: clinical and radiographic criteria at week 24, 52 and 104. Results: 23 trials (6358 patients), including seven bDMARDs and one other molecule: Anbainuo (anti-TNF-R). No study satisfied our search criteria for anakinra, certolizumab and infliximab. Compared to bDMARD monotherapy, combination therapy gives a better ACR 20 at 24 weeks (RR: 0.88 (0.84–0.94)) in fixed and random effect models, and this result is sustained at 52 and 104 weeks. The results were mostly similar for all other outcomes without increasing the risk of adverse effects. Conclusion: This meta-analysis confirms the superiority of combination therapy over monotherapy in rheumatoid arthritis, in accordance to the usual guidelines.

## 1. Introduction

Rheumatoid arthritis (RA) is the most common inflammatory rheumatism in adults [1]. The EULAR and French recommendations stipulate that methotrexate (MTX) should be started as soon as possible after the diagnosis of established RA [1,2]. If remission or low disease activity is not achieved after six months of conventional synthetic DMARDs (CsDMARDs) in patients without factors associated with a poor prognosis, treatment with another CsDMARD may be considered. By contrast, in the presence of a poor prognosis factor, biological treatment should be considered in association with the CsDMARD previously used [3]. In total, ten biological agents (bDMARDs) have been approved for RA treatment. Among them, adalimumab, certolizumab and etanercept have also been approved for use in monotherapy, as have abatacept, anakinra, tocilizumab and sarilumab. New strategies for RA treatment based on the inhibition of Janus kinase (JAK) pathways have been developed, but are not reviewed here. Many RA patients find it difficult to adhere to their CsDMARD prescription because of intolerance or contraindications [4,5,6,7,8,9,10]. It is, therefore, important to evaluate the benefits and harm associated with the use of biological agents in monotherapy. We conducted a systematic review of the literature and a meta-analysis, to update the available evidence already established [11] comparing the use of bDMARD and CsDMARD combination therapy with the use of biotherapy in monotherapy in patients with rheumatoid arthritis.

## 2. Materials and Methods

A PICOS design (Participants, Interventions, Comparisons and Outcomes) was used for the search strategy. The study selection, assessment of eligibility criteria, data extraction and statistical analyses were performed with a predefined protocol [12]. The reporting of the systematic review and meta- analysis conforms to the PRISMA (Preferred Reporting Items for Systematic reviews and Meta-analyses) statement [13].

### 2.1. Literature Search 

CENTRAL, EMBASE and MEDLINE were used to identify published reports. Additional randomized controlled trials (RCTs) identified in relevant systematic reviews not retrieved through the electronic databases were then collated.

### 2.2. Trial Selection 

Articles were screened independently by two authors of the review (C.D. and L.F.X.) for inclusion on the basis of their title, abstract and full text if necessary. Disagreements were resolved by consensus or through discussion with a third author (P.H.). Search results were limited to randomized controlled trials (RCTs) with two arms. We included open-label trials in our qualitative and quantitative analysis and performed a meta-regression analysis to determine whether the inclusion of these studies with a lower grade of recommendation modified our findings. We did not include trials for which a full text in English was not available and trials not reporting American College of Rheumatology (ACR) responses. We chose to stop studying inclusion after 104 weeks of follow-up. 

### 2.3. Participants

Adults (>18 years) with RA, according to the 1987 or 2010 classification criteria.

### 2.4. Types of Interventions 

Biologics used alone compared to biologics used in combination with a conventional synthetic DMARD.

### 2.5. Outcome Measures 

We used all the available data published in the selected studies for the meta-analysis. We decided a priori to use the outcome assessment at 24 weeks, 52 and 104 weeks, to determine whether early outcomes were sustained over time. Our major outcome was the ACR 20 response criteria at 24 weeks. The secondary outcomes were: the ACR 20 criteria at 52 and 104 weeks, the ACR 50, 70, 90 response criteria, the DAS 28 remission score (including C-reactive protein (CRP) concentration or erythrocyte sedimentation rate (ESR)), the proportion of Van der Heide-modified Sharp’s scores (mTSS) non-progressor (≤0.5), the proportion of patients withdrawing from the study due to adverse events and for lack of efficacy, improvement in the Health Assessment Questionnaire (HAQ) score > 0.22 and remission according to the Clinical Disease Activity Index (CDAI) and Simple Disease Activity Index (SDAI) scores. Concerning tolerance, we assessed adverse events, serious adverse events, infections, serious infections, cancers and tuberculosis.

### 2.6. Data Collection and Handling of Missing Data 

Data from the trials were independently extracted by two abstractors (C.D. and F.X.L.). We obtained additional information from the online Supplementary Materials of the original RCTs when necessary. For graphic data, we used WebPlotDigitizer**-4.2 copyright 2010–2019 Ankit Rohatgi** for digital data extraction. Several studies [14,15,16,17] have shown the tool’s reliability and validity to extract data from single-case graphs. 

### 2.7. Risk of Bias

**Figure 1:**
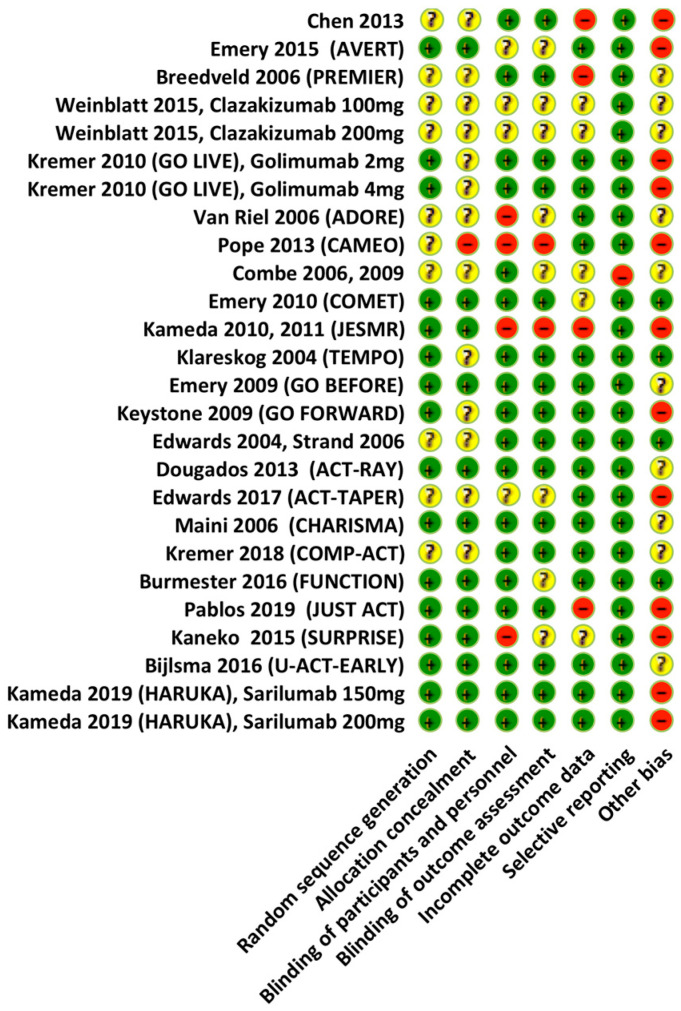
Assessment of the risk of bias. The Cochrane Collaboration tool used for randomized trials.

We assessed the risk of bias for each trial included, using the Cochrane ‘Risk of bias’ tool and the following criteria: selection bias, performance bias, detection bias, attrition bias and reporting bias [18]. The risk of bias has been classified as: ‘low’, ‘high’ or ‘unclear’ (due to either a lack of information or uncertainty over the potential for bias). The GRADE score reflects the extent to which we are convinced that the actual effect is close to that estimated in the meta-analysis (Figure 1).

### 2.8. Statistical Analysis

We performed meta-analyses with fixed and random effects models in R version 3.6.1 (5 July 2019) Copyright © 2022 The R Foundation for Statistical Computing. The relative risk (RR) was the metric of choice for binary outcomes, and the mean difference (MD) or the standardized mean difference (SMD) was used for quantitative variables. Inverse variance weighting was used for the pooling of studies [19]. We used the DerSimonian–Laird method to estimate the variance between studies [20]. Between-study heterogeneity was assessed with the Q-test, considering a *p*-value < 0.05 to be statistically significant. The I² statistic was calculated to quantify the residual heterogeneity, ranging from 0 to 100% [21]. A leave-one-out method was used to identify outlying studies responsible for heterogeneity. Sensitivity analyses were conducted by meta-regression. The criteria included in the meta-regression analysis included: dose titration allowed in the study protocol, mean duration of disease, history of CsDMARD and bDMARD use before inclusion, presence or absence of a disease stabilization phase before randomization, positivity for RF and/or ACPA, severity on the DAS at inclusion, authorization of corticosteroid therapy use during the study and blinding throughout the study. Publication bias was evaluated by a graphical method, with a rank correlation test for funnel plot asymmetry [22]. 

## 3. Results

### 3.1. Study Selection Process

**Figure 2:**
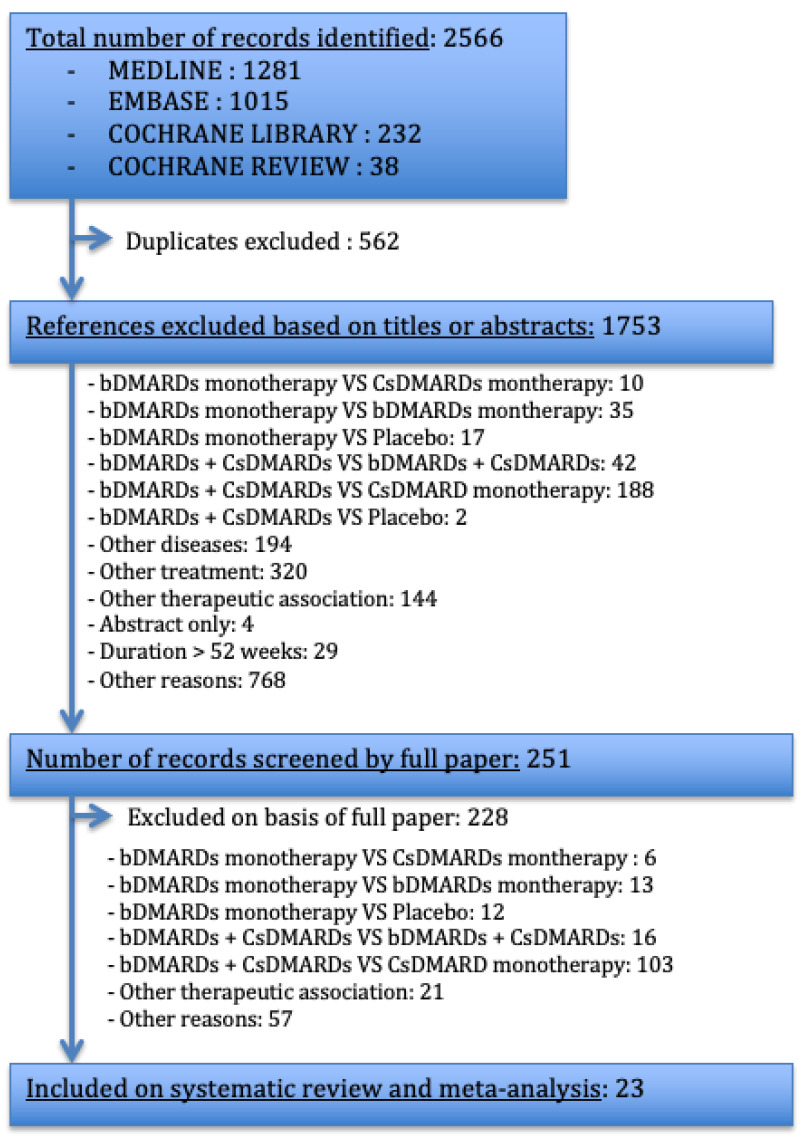
Flow diagram for study inclusion/exclusion. CsDMARDs: Conventional synthetic disease-modifying anti-rheumatic drugs; bDMARDs: Biological disease-modifying anti-rheumatic drugs; VS: versus.

We identified 2566 publications: 1281 from PUBMED, 1015 from EMBASE, 232 from the Cochrane Library and 38 Cochrane reviews (Figure 2). We retained 23 articles, corresponding to 6358 patients. Of these studies, one focused on abatacept [23], one on adalimumab [24], six on etanercept [25,26,27,28,29,30,31,32], one on a similar molecule of etanercept, abainuo [33], three on golimumab [34,35,36,37,38], one on rituximab [39,40], eight on tocilizumab [41,42,43,44,45,46,47,48,49,50], one on sarilumab [51] and one on clazakizumab [52]. Of these studies, four had open-label designs [29,30,32,50]. In the COMET [28], COMP-ACT [48] and JUST-ACT [47] studies, the participants were randomized into the two arms of interest after an initial period of 52 weeks, 24 weeks and 16 weeks, respectively, during which all patients received a combination of bDMARDs + CsDMARDs. In the ACT-TAPER [4] study, patients achieving a good/moderate EULAR response were randomized to a double-blind MTX taper arm, in which the MTX dose was tapered to 5 mg at 16 weeks, with complete withdrawal of MTX at 24 weeks, or to a stable MTX dose arm. We defined the day of inclusion, D0, for this study as the day on which methotrexate was completely withdrawn. We have not identified any studies satisfying our search criteria for anakinra, certolizumab or infliximab.

### 3.2. Study Characteristics 

The characteristics of the studies included in the meta-analysis are displayed in Table 1. Regarding the history of CsDMARD use, three studies included only patients who had never used CsDMARDs (13%), 16 included patients not naïve for CsDMARD use (69.6%) and four were not selective on the basis of these criteria (17.4%). All but two of the studies used MTX as the CsDMARD. For the history of bDMARD use, nine studies included only patients who had never used bDMARDs (39.1%), eight included patients with a possible history of use before inclusion (34.8%) and five included patients not naïve for bDMARD treatment (21.7%). Of the studies, eight included (26.1%) planned treatment adjustment during the trial a priori, with a rescue treatment administered if the main endpoint was not reached within the time allowed. 

### 3.3. Principal Characteristics of the Patients

More than 75% of the patients were women, and the mean age was 55 years (range: 45.4 ± 11.9 to 63.3 ± 10.6 years). Disease duration ranged from 26 days to 12 years, and most of the patients for whom the information was available had tested positive for autoantibodies (RF and/or ACPA). The DAS 28 score at baseline ranged from 2.6 to 6.8, and the mTSS score ranged from 0 ± 0 to 39.6 ± 56.1.

### 3.4. Primary Efficacy Endpoint: ACR 20 at 24 Weeks

**Figure 3:**
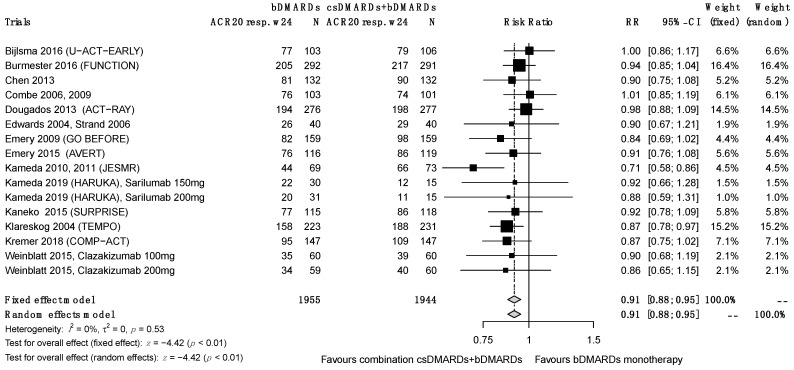
ACR 20 responses at week 24.

The ACR 20 results at 24 weeks were reported for 16 studies. Using a random effects model, we found that the therapeutic combination performed significantly better for this endpoint, RR = 0.88 (0.83; 0.93), despite significant heterogeneity between studies (I^2^ = 46%, τ^2^ = 0.0068, *p* = 0.02) (Figure 3). We made the results more consistent by performing a sensitivity analysis and a funnel plot asymmetry test. The remaining 14 studies showed that significantly better results for this endpoint were obtained with the combination treatment (RR = 0.91 (0.88; 0.95); I_2_ = 0%, τ^2^ = 0, *p* = 0.53). We decided a priori to include open-label studies in the quantitative analysis and to use the qualitative analysis to check whether this decision had any effect on the results. Consistent with the τau^2^ test results, we can conclude that the inclusion of SUPRISE, ADORE and JESMR with an open-label design, despite their lower grade of recommendation, had no effect on the results of the analysis.

### 3.5. Other Endpoints

#### 3.5.1. ACR Reponses

With fourteen studies providing ACR 20 results at 52 weeks, the overall result obtained with the random effects model was significantly in favor of the therapeutic combination (RR= 0.90 (0.84; 0.97)). However, it was not possible to have confidence in the results, due to the degree of heterogeneity (I^2^ = 64%, τ^2^ = 0.0116, *p* < 0.01). After controlling for heterogeneity, we selected 11 studies. The results were also in favor of the combination treatment (RR = 0.94 (0.90; 0.98), I^2^ = 7%, τ^2^ = 0.0004, *p* = 0.38). The ACR 20 results at 104 weeks were available for eight studies. The results were significantly in favor of the combination treatment both before (RR = 0.89 (0.84; 0.94), I^2^ = 66%, τ^2^ = 0.0129, *p* < 0.01) and after the sensitivity analysis (RR = 0.92 (0.87; 0.98)) (I² = 0%, τ² = 0, *p* = 0.42). The ACR 50 scores were significantly in favor of the combination treatment and were not affected by sensitivity testing (RR = 0.81 (0.76; 0.87), I^2^ = 0%, τ^2^ = 0, *p* = 0.77) at 24 weeks, (RR = 0.89 (0.82; 0.97), I^2^ = 10%, τ^2^ = 0.0019, *p* = 0.35) at 52 weeks and (RR = 0.84 (0.77; 0.93), I^2^ = 14%, τ^2^ = 0.0022, *p* = 0.32) at 104 weeks. The same was true for the ACR 70 score at weeks 24 and 52 (RR = 0.76 (0.68; 0.85), I^2^ = 10%, τ^2^ = 0.0055, *p* = 0.33) at 24 weeks, (RR = 0.81 (0.73; 0.90), I^2^ = 16%, τ^2^ = 0.0063, *p* = 0.28) at 52 weeks. At 104 weeks, the ACR 70 data were significantly in favor of the combination treatment in the analysis (RR = 0.77 (0.64; 0.93), I^2^ = 61%, τ^2^ = 0.00393, *p* = 0.01), but were not significant after sensitivity analysis (RR = 0.89 (0.78; 1.01), I^2^ = 0%, τ^2^ = 0, *p* = 0.84). Overall, five studies reported ACR 90 scores at 24 weeks, showing these results to be significantly in favor of the combination treatment (RR = 0.64 (0.44; 0.93), heterogeneity: I^2^ = 0%, τ^2^ = 0, *p* = 0.70). At 52 weeks, the ACR 90 data were not significantly in favor of either therapeutic strategy, even after sensitivity analysis (RR = 0.88 (0.65; 1.19), I^2^ = 0%, τ^2^ = 0, *p* = 0.84). Only two studies reported ACR 90 scores at 104 weeks; the lack of data did not allow for reliable analyses.

#### 3.5.2. Remission According to DAS 28 (Using ESR or CRP)

**Figure 4:**
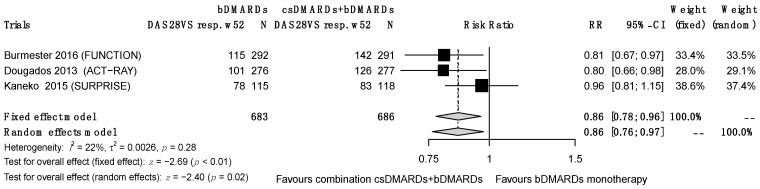
DAS 28–ESR remission at 52 weeks.

At 24 weeks, remission according to DAS 28 (<2.6) was reported, based on CRP in six RCTs and ESR in eight RCTs. For the DAS 28–CRP remission scores, the objective was achieved significantly more frequently for the combined treatment group at weeks 24 and 52 (RR = 0.66 (0.56–0.77), I^2^ = 0%, τ² = 0.00, *p* = 0.70) at 24 weeks and (RR = 0.73 (0.63–0.85), I^2^ = 0%, τ^2^ = 0.00, *p* = 0.92) at 52 weeks. Only two studies provided results at 104 weeks. For the DAS 28–ESR remission scores, the initial data at 24 weeks showed an I^2^ value of 0% for heterogeneity, but the funnel plot asymmetry test revealed a publication bias for the GO-FORWARD study, so we excluded this study from the final analysis. The objective was significantly more frequently achieved in the association group at weeks 24 and 52 (at 24 weeks, RR = 0.87 (0.80–0.95), I^2^ = 0%, τ^2^ = 0, *p* = 0.90 (Figure 4); at 52 weeks, RR = 0.86 (0.76–0.97), I^2^ = 22%, τ^2^ = 0.0026, *p* = 0.28), but the results ceased to be significant at 104 weeks (RR = 0.83 (0.83–1.05), I^2^ = 0%, τ^2^ = 0.00, *p* = 0.89). Note that the results at 52 weeks were initially non-significant but became so after sensitivity analysis.

#### 3.5.3. HAQ, CDAI and SDAI Scores

In total, nine RCTs reported an improvement in HAQ ≥ 0.22 and showed an absence of significance for either arm of the study at 24 weeks (RR = 0.90 (0.80; 1.01), I^2^ = 0%, τ^2^ = 0.00, *p* = 0.49), but a significant difference emerged from 52 weeks onwards in favor of the combination therapy (RR= 0.83 (0.75; 0.92), I² = 4%, τ^2^ = 0.0005, *p* = 0.37 at 52 weeks; RR= 0.89 (0.83; 0.96), I^2^ = 17%, τ^2^ = 0.0012, *p* = 0.31 at 104 weeks). Similar results were obtained for CDAI remission (<2.8) at 24, 52 and 104 weeks and SDAI remission (<3.3) at 24 and 52 weeks after sensitivity analysis (no results available at 104 weeks). 

#### 3.5.4. Subgroup Meta-Analysis 

All our results show an RR close to 1, meaning that the combination therapy was not much more effective than the biological agent used as a monotherapy. We decided to perform a subgroup analysis by successively removing etanercept, tocilizumab and then both molecules from the analysis to try to explain these results. We found that removing tocilizumab resulted in a decrease in RR, whereas removing etanercept did not appear to change the results. This sub-analysis suggests that tocilizumab appears to be more effective as a single agent than other available biotherapies. 

#### 3.5.5. Structural Progression

**Figure 5:**
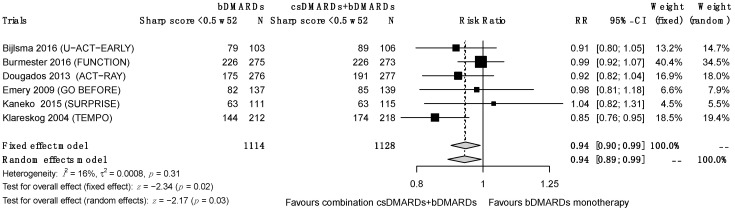
Sharp remission at 52 weeks.

A total of eleven RCTs reported X-ray progression based on mTSS <0 or <0.5 at weeks 24, 52 and/or 104. A significantly lower progression was observed for the combination treatment from 52 weeks (Figure 5) and remained at 104 weeks before and after sensitivity analysis; the results at 24 weeks were not significant (RR = 0.98 (0.91; 1.04), I^2^ = 6%, τ^2^ = 0.0003, *p* = 0.35 at 24 weeks; RR = 0.94 (0.89; 0.99), I^2^ = 16%, τ^2^ = 0.0008, *p* = 0.31 at 52 weeks; RR = 0.92 (0.87; 0.98), I^2^ = 12%, τ^2^ = 0.0008, *p* = 0.34).

#### 3.5.6. Discontinuation Due to a Lack of Efficacy

The rate of discontinuation due to lack of efficacy did not differ between groups up to 52 weeks (RR = 1.74 (0.92; 3.29) with I^2^ = 0%, τ^2^ = 0, *p* = 0.59 at 24 weeks; RR = 1.39 (0.88; 2.18) with I^2^ = 6%, τ^2^ = 0.0241, *p* = 0.39 at 52 weeks). However, an increase in the number of discontinuations due to a lack of efficacy was reported at 104 weeks for the biotherapy monotherapy group (RR= 2.83 (1.82; 4.41) with I^2^ = 19%, τ^2^ = 0.0398, *p* = 0.30 at 104 weeks). 

#### 3.5.7. Toxicity

The heterogeneity of the toxicity data made it impossible to perform a reliable statistical analysis. Nevertheless, we were able to compare study outputs for discontinuation due to adverse events. We found no advantage for either group in terms of the number of discontinuations: RR = 0.73 (0.53; 1.01) with I^2^ = 0% and τ^2^ = 0, *p* = 0.70 at 24 weeks; RR= 0.95 (0.70; 1.31) with I² = 40%, τ^2^ = 0.1151, *p* = 0.07 at 52 weeks; and RR = 0.84 (0.67; 1.05) with I^2^ = 0%, τ^2^ = 0, *p* = 0.53 at 104 weeks. The I² value for heterogeneity obtained at 52 weeks was >30%. We decided to retain this result because significance did not differ before and after sensitivity analysis, and a cutoff of 40% has been reported to be acceptable [54]. 

## 4. Discussion

Through this systematic review and meta-analysis, we aimed to compare the use of biological agents in monotherapy and in association with a CsDMARD. Many meta-analyses have compared studies of different therapeutic combinations (for example, biotherapies in monotherapy versus CsDMARDs or placebo), making it difficult to extrapolate results to our arms of interest [55,56,57,58,59]. 

This is the second systematic review comparing the value of adding MTX to bDMARD treatment with bDMARD monotherapy. Our work confirms the work of Tarp and al. [11], with the difference that our study is more recent, which allowed us to include a larger number of randomized trials and, therefore, patients.

We found that the combination treatment was more effective than monotherapy, as shown by the main endpoint, ACR 20 at 24 weeks. The results are similar for the other endpoints, with, for some, a loss of efficacy at 104 weeks, possibly with a loss of power of the study. The modified Sharp’s score was significantly in favor of the combined treatment from 52 weeks onwards. At 24 weeks, the duration of exposure may not have been sufficiently long to distinguish between the two study arms considered. It is interesting to note that the PREMIER study [24] was a source of great heterogeneity for several of the variables studied with no obvious cause found. Our results are comparable with French and international recommendations [1,2]. 

The purpose of this meta-analysis was not to compare the different bDMARDs between each other. Nevertheless, we could observe that all our results showed an RR close to 1, showing little difference in clinical efficacy between groups. The subgroup analysis showed that excluding tocilizumab from the analysis decreased this RR, suggesting that tocilizumab is probably the most effective single-agent biologic. These results are consistent with the literature. Tarp et al. [60] have shown that most biological agents are effective in monotherapy, with an advantage for etanercept and tocilizumab supported by other network meta-analyses [57,59,61]. Some studies about IL-6 receptor blockers in monotherapy have shown that tocilizumab monotherapy yields response rates close to those obtained in combination with MTX in randomized studies and cohorts [62,63]. The ADACTA and MONARCH studies have shown tocilizumab and sarilumab to be superior to adalimumab in monotherapy [64,65]. In the TOCERRA registry [63], the therapeutic efficacy and maintenance of tocilizumab monotherapy are similar to those of the anti-TNF agents associated with MTX. 

Finally, structural damage was not studied for all the biological treatments included in our meta-analysis. Tarp et al. [11] obtained identical results to those reported here and, after a subgroup analysis, no structural differences were found.

We found no difference in terms of safety between the two treatment arms, essentially due to the heterogeneity of the data collection. However, we were able to show that there was no difference between the study arms in terms of the rate of treatment discontinuation due to adverse events. With regard to the risk of infection, Singh et al. [66] showed that, in patients treated with CsDMARDs, the median annual absolute risk of infection was 2%, or 20 per 1000 treated patients per year, whereas there was an increase to 6 per 1000 patients treated with bDMARDs in combination with a CsDMARD, with a significant difference. Ramiro et al. [67] confirmed that patients on bDMARDs (both anti-TNF and no anti-TNF agents) had a higher risk of serious infections than patients on CsDMARDs, and that there was generally no difference between bDMARDs. They also investigated the occurrence of different cancers after exposure to biologics. Relative to both the general population and patients on CsDMARDs, patients on bDMARDs had no higher risk of individual solid cancers or of lymphoma. By contrast, non-melanoma skin cancer may occur more frequently in patients on bDMARDs than in the general population (HR 1.7), but the risk in these patients is no higher than that in patients treated with CsDMARDs. One study with a low risk of bias showed that patients on bDMARDs may have a higher risk of melanoma than patients on CsDMARDs (HR 1.5, 95% CI 1.0 to 2.2) [68].

Nevertheless, our study has several limitations. First, as with all systematic literature reviews, this study is subject to certain publication and selection biases. Second, there was some heterogeneity between these studies. The variables tested with the sensitivity analysis did not significantly influence the results of the meta-analysis. In addition, few studies have used CsDMARDs other than methotrexate, limiting the extrapolation of results for leflunomide or sulfasalazine. We also chose to consult the data collected at weeks 24, 52 and 104; when data for these time points were not available, other time points were used. Comparisons at different time points may limit the interpretation of our results, as may not having been able to contact the authors to recover missing data. 

## 5. Conclusions

Our meta-analysis confirms the results of a previous one, but with updated research and a larger number of studies included. The results indicate that the combination therapy of a biological agent with CsDMARDs is more effective than monotherapy and should be preferred in uncontrolled RA, in accordance with the usual guidelines. MTX should be switched to another CsDMARD in the case of contraindication or intolerance.

## Figures and Tables

**Table 1:** Study characteristics.

Studies	Follow-Up	CsDMARD History	WO Period	bDMARD History	WO Period	RA Duration	Treatment	Doses (mg)	Dose Adjustment Defined a Priori	N
**Anbainuo,** **Chen, 2013 [33]**	24 weeks	Naive	/	Naive	/	ND	Abainuo + MTX Abainuo	Abainuo: 25 mg SC 2x/Week MTX: 10–15 mg/Week PO	No	N = 132 N = 132
**Abatacept (AVERT), Emery, 2015 [23]**	48 weeks	Naive: MTX-naive or received MTX (≤10 mg/week) for ≤4 weeks	MTX 1 month	Naive	/	<2 years	ABA + MTX ABA	ABA: 125 mg SC/Week MTX: 7.5–20 mg/Week PO	No	N = 119 N = 116
**Adalimumab (PREMIER), Breedveld, 2006 [24]**	48/104 weeks	Naive or not: MTX, cyclophosphamid, CYC, AZA, or 2 other CsDMARDs were excluded	4 weeks	Naive	/	<3 years	ADA + MTX ADA	ADA: 40 mg SC/2 weeks MTX: 7.5–20 mg/Week PO	Increased dosing with ADA/placebo to weekly if ACR 20 not achieved in 2 consecutive visits after week 16.	N = 268 N = 274
**Clazakizumab, Weinblatt, 2015 [53]**	24 weeks	Non naive: MTX failure (>3 months treatment)	/	Naive	/	>16 weeks	CLZ + MTX CLZ	CLZ: 100 mg SC/4 wks MTX: 10–22 mg/Week PO	If <20% reduction SJC/TJC: receive open-label CLZ 200 mg SC/4 wk + MTX	N = 60 N = 60
**Etanercept, (ADORE), van Riel, 2006 [32]**	16 weeks	Non naive: MTX >12.5 mg/week for >3 months	12 weeks	Naive	/	ND	ETN + MTX ETN	ETN: 25 mg SC 2x/Week MTX: >12.5 mg/week PO or SC	No	N = 155 N = 159
**Etanercept, (CAMEO), Pope, 2013 [29]**	24/104 weeks	Non naive: MTX therapy for >12 weeks	/	Non naive: ETN + MTX for 6 months	/	> 6 months	ETN + MTX ETN	ETN: 50 mg SC/Week MTX: ≥15 mg/week	No	N = 107 N = 98
**Etanercept, Combe, 2006 [25]**	24/48/104 weeks	Non naive: SSZ for >4 months	Other than SSZ: 3 month	Naive or not: ineligible if they had received ETN or other TNF antagonists	bDMARDs or CTX: 6 months	<20 years	ETN + SSZ ETN	ETN: 25 mg SC 2x/Week SSZ: 2–2.5–3 g/day PO	No	N = 101 N = 103
**Etanercept (COMET), Emery, 2010 [28]**	52 weeks	Non naive: ETN + MTX for 52 weeks before new randomization.	No	Non naive: ETN + MTX during 52 weeks before new randomization.	No	4 months until 2 years	ETN + MTX ETN	ETN: 25 mg SC 2x/week MTX 7.5–20 mg/Week PO	No	N = 111 N = 111
**Etanercept, (JESMR), Kameda, 2010 and 2011 [30,31]**	24/52 weeks	Non naive: MTX 6 mg/week for >3 months	/	Naive	/	ND	ETN + MTX ETN	ETN: 25 mg SC 2x/Week MTX: 6–8 mg/week	No	N = 76 N = 71
**Etanercept (TEMPO), Klareskog, 2004 [27]**	24/52/ 104 weeks	CsDMARD non naive, but MTX naive or not	MTX 6 month	Naive or not: Ineligible if previously received ETN or other TNF antagonists.	ISD: 6 months bDMARD: 3 months	6 months until 20 years.	ETN + MTX ETN	ETN: 25 mg SC 2x/week MTX: 7.5–20 mg/Week PO	No	N = 231 N = 223
**Golimumab (GO BEFORE), Emery, 2009 [35]**	24/52/104 weeks	Naive or not: had not received more than 3 weekly doses of oral MTX	/	Naive or not: IFX, ETN, ADA, RTX, NTZ, or cytotoxic agents, and alkylating agents, were excluded	ANK: 4 weeks alefacept/ efalizumab: 3 months, other: 5 half-lives	3 months until 3 years	GOL + MTX GOL	GOL: 100 mg SC/4 weeks MTX: 10–20 mg/Week PO	If <20% improvement from baseline SJC/TJC entered early escape any time after week 24.	N = 159 N = 159
**Golimumab (GO** **FORWARD), Keystone, 2009 [38]**	24/52/104 weeks	Non naive: had been receiving a stable dose of MTX 15–25 mg/week for at least 4 weeks	Other than MTX 4 weeks	Naive or not: excluded if used anti- TNF agent, RTX, NTZ or cytotoxic agents	ANK: 4 weeks alefacept efalizumab: 3 months	NR	GOL + MTX GOL	GOL: 100 mg SC/4 weeks MTX: 15–20 mg/week PO	If <20% improvement from baseline TJC/SJC escape any time after week 24.	N = 89 N = 133
**Golimumab (GO LIVE), Kremer, 2010 [37]**	24/48 weeks	Non naive: MTX for >3 months	/	Naive or not: limited to 20% of the study population. (Excluded if RTX, ABA, or NTZ).	IFX, alefa- Cept/efalizumab: 3 months, ETN/ADA 2 monthsANK/ABA/NTZ. 4 weeks	<8 years	GOL 2 mg/kg + MTX GOL 4 mg/kg + MTX GOL 2 mg/kg GOL 4 mg/kg	GOL: 2 mg/kg OR 4 mg/kg IV/12 weeks MTX: 15 mg/Week PO	At weeks 16 and 24, patients with <20% improvement from baseline in both the SJC and TJC entered early escape and dose regimen	N = 128 N = 129 N = 129 N = 128
**Rituximab, Edwards, 2004, Strand, 2006 [40,41]**	24/48/104 weeks	Non naive: had failed 1–5 CsDMARDs and MTX with treatment for >16 weeks	/	ND	/	ND	RTX + MTX RTX	RTX: 1000 mg IV on days 1 and 15 all 6 months MTX: 12.5–15 mg/Week PO	No	N = 40 N = 40
**Sarilumab, (HARUKA)Kameda, 2019 [52]**	24/52 weeks	Naive or not: -monotherapy: CsDMARDs naive -combination: CsDMARDs non naive	/	Naive or not	CYC, MFMAZA, CTX, bDMARD: 4–12 weeks	ND	SLM 150 mg + non-MTX CsDMARDs SLM 200 mg + non-MTX CsDMARDs SLM 150 mg SLM 200 mg	SLM 150 or 200 mg/2 Weeks SC	No	N = 15 N = 15 N = 30 N = 31
**Tocilizumab (ACT RAY), Dougados, 2013 [42]**	24/52/104 weeks	Non naive: MTX for at least 12 weeks	LEF: 3 moth Other 1 month	Naive or not	bDMARD 1 month	ND	TCZ + MTX TCZ	TCZ: 8 mg/kg IV/4 weeks MTX: 15–20 mg/Week PO	At week 24, if DAS28 > 3.2; an open-label CsDMARD was added. At week 36, if DAS28 > 3.2, an additional CsDMARD added.	N = 277 N = 276
**Tocilizumab (ACT-TAPER), Edwards, 2017 [50]**	24 weeks	Non naive: had inadequately responded to 2 CsDMARDs, including MTX.	/	Non naive	Had have TCZ 8 mg/kg/4 weeks for 24 weeks	ND	TCZ + MTX stable dose TCZ + MTX Tapering dose	TCZ: 8 mg/kg IV/4 weeks MTX stable dose: 10–15 mg/Week MTX tapering dose S24 to S40: 5 mg /week; S40 to S48: 0 mg.	No	N = 136 N = 136
**Tocilizumab (CHARISMA), Maini, 2006 [54]**	16/20 weeks	Non naive: MTX failure >6 months of treatment	LEF: 6 months Other 4 weeks	Naive or not	anti-TNF agents: 12 weeks	ND	TCZ + MTX TCZ	TCZ: 8 mg/kg IV/4 weeks MTX: 10–25 mg/Week PO	No	N = 50 N = 52
**Tocilizumab (COMP-ACT), Kremer, 2018 [49]**	24 weeks	Non naive: TCZ + MTX during 24 weeks before new randomization.	/	Non naive: TCZ + MTX for 24 weeks before new randomization.	/	ND	TCZ + MTX TCZ	TCZ: 162 mg/week (≥100 kg) or /2 weeks (<100 kg) MTX: >15 mg/week PO	No	N = 147 N = 147
**Tocilizumab (FUNCTION), Burmester, 2016 [45]**	24/52/104 weeks	CsDMARD-naive or not but MTX-naive	/	Naive	/	<2 years	TCZ + MTX TCZ	TCZ:8 mg/kg IV/4 wks MTX: 7.5–20 mg/Week PO	No	N = 291 N = 292
**Tocilizumab (JUST ACT), Pablos, 2019 [48]**	12 weeks	Non naive: TCZ + MTX 16 weeks before randomization.	/	Non naive: TCZ + MTX 16 weeks before randomization.	/	NR	TCZ + MTX TCZ	TCZ: 8 mg/kg IV/4 wks MTX: >15 mg/Wek PO	No	N = 83 N = 82
**Tocilizumab, (SURPRISE), Kaneko, 2015 [51]**	24/52/104 weeks	Non naive: MTX ≥6 mg/week for at least 8 weeks	LEF: 12 weeks, other: 8 weeks	Naive	Tacrolimus: 4 weeks	<10 years	TCZ + MTX TCZ	TCZ: 8 mg/ kg IV/4 wks MTX: >6 mg/Week PO	No	N = 118 N = 115
**Tocilizumab (U-ACT-EARLY), Bijlsma, 2016 [46]**	24/52/104 weeks	Naive	/	Naive	/	<1 year	TCZ + MTX TCZ	TCZ:8 mg/ kg IV/4 wks MTX: 10–30 mg/Week PO	No	N = 106 N = 103

ND: not disclosed; N: number; IV: intravenous; IM: intramuscular; PO: per os; SC: subcutaneous; wks: weeks; mths: months; WO: wash out; min: minimum; ACR 20: American college of Rheumatology 20; SJC: swollen joint count; TJC: tender joint count; sDMARDs: conventional disease-modifying antirheumatic drugs; bDMARDs: biologic disease-modifying antirheumatic drugs; ABA: abatacept; ADA: adalimumab; ANK: anakinra; AZA: azathioprine; CLZ: clazakizumab; CTX: cyclophosphamide; CYC: ciclosporine; CZP: certolizumab pegol; ETN: etanercept; GOL: golimumab; HCQ: hydroxychloroquine; IFX: infliximab; ISD: immunosuppressive drug; LEF: leflunomide; MMF: mycophenolate; MTX: methotrexate; NTZ: natalizumab; RTX: rituximab; SLM: sarilumab; SSZ: sulfasalazine; TCZ: tocilizumab.

## Data Availability

For the unpublished data, we collected data on the EULAR response criteria, as well as the variation over time of ACR-N, DAS 28-ESR and DAS28-CRP, total sharp score, erosions and joint-space scores, HAQ-DI score, as well as the variation of ESR, CRP, number of painful and swollen joints, VAS pain, patient global VAS and physician global VAS. The information is available on request by e-mail from Célia Delpech.

## Supplementary Materials

The following supporting information can be downloaded at: https://www.mdpi.com/article/10.3390/jcm12010286/s1. Figure S1: Original RCTs.
