# Supplementary material for: Comparison of Biological Agent Monotherapy and Associations Including Disease-Modifying Antirheumatic Drugs for Rheumatoid Arthritis: Literature Review and Meta-Analysis of Randomized Trials
Source: J Clin Med. 2022 Dec 29;12(1):286. doi: 10.3390/jcm12010286 (PMC9821556; doi:10.3390/jcm12010286)
Supplement: Supplementary file 1 [file jcm-12-00286-s001.zip › jcm-2065612-supplementary.pdf]

COMPARISON OF BIOLOGICAL AGENT MONOTHERAPY AND ASSOCIATIONS INCLUDING DISEASE-MODIFYING ANTIRHEUMATIC DRUGS FOR RHEUMATOID ARTHRITIS: LITERATURE REVIEW AND META-ANALYSIS OF RANDOMIZED TRIALS

Célia Delpech, François-Xavier Laborne, Pascal Hilliquin
CHSF, Corbeil-Essones, France

Background
Biologic disease-modifying antirheumatic drugs (bDMARDs) extend the treatment choices for rheumatoid arthritis (RA) patients with suboptimal response or intolerance to conventional synthetic DMARDs (CsDMARDs).

Objective : To compare the efficacy and safety of the individual biological agents used in monotherapy in patients with RA than the combination therapy strategy with CsDMARD + bDMARD.

Methods:
We used The Cochrane Central Register of Controlled Trials (CENTRAL), EMBASE, and MEDLINE in order to carry out our research, for published reports from inception of each database through December 2019.
Search results were limited to randomised controlled trials (RCTs), with our two arms : biological agent in monotherapy and combination strategy (with any CsDMARDs).

Major outcome: ACR 20 reponse criteria at 24 week.
Secondary outcomes: the ACR 20 at 52 weeks, ACR 50, 70, 90 reponse criteria, the DAS 28 remission (with CRP and/or ESR), the proportion of non progressors evaluated by the modified Sharp's score, the proportion of patients who withdraw from the study due to adverse events, the proportion of patients who withdraw from the study due to lack of efficacy, the HAQ improvement > 0,22, CDAI and SDAI remission at weeks 24 and 52 if the data were available.

To estimate the relative efficacy of treatments whilst preserving the randomized comparisons within each trial, a Bayesian network meta-analysis was conducted in R (version 3.6.1) using fixed and random-effects

Results:
The analysis comprises 23 trials (6404 patients), including 7 biological agents approved for RA (abatacept, adalimumab, etanercept, golimumab, rituximab, sarilumab and tocilizumab) as well as two other molecules : Clazakizumab, a humanized monoclonal antibody that binds to the interleukin-6 (IL-6) cytokine and Anbainuo, a recombinant human TNFRII:Fc fusion protein. No study satisfied our search criteria for anakinra, certolizumab and infliximab.

Compared to combination strategy, bDMARD monotherapy has less probability to give ACR20 response at 24 weeks (RR : 0,92 [0,89 – 0,96]) in fixed or random effect model.
Result is similar at 52 weeks (RR : 0,94 [0,89 – 0,99]).
<u>For all other outcome mesures</u>, we can see an increased of ACR50–70 and 90 responses, an improve of the DAS 28 remission score, an increase of the proportion of sharp's score non progressors (<0,5) as well as a decrease of withdrawals for inefficacy without increase of withdrawals for toxicity.

Conclusion:
Evidence from this meta-analysis suggests that combinaison strategy with bDMARD+CsDMARD remains the preferred treatment and seems more effective than the use of biologics in monotherapy.
The interest from this point of view is to sensitize prescribers to the use of other CsDMARDs when there is a contraindication or intolerance to MTX, but also to make patients aware of their treatment by reaffirming the superiority of the association compared to the use of biological agent alone.

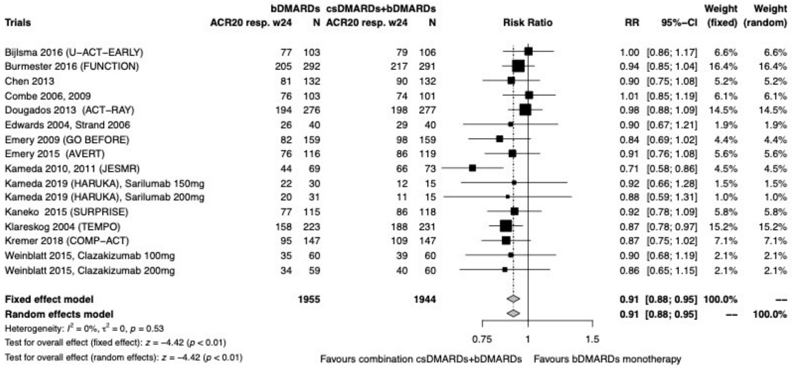

Trials	bDMARDs ACR20 resp. w24	N	csDMARDs+bDMARDs ACR20 resp. w24	N	RR	95%–CI	Weight (fixed)	Weight (random)
Bijlsma 2016 (U–ACT–EARLY)	77	103	79	106	1.00	[0.86; 1.17]	6.6%	6.6%
Burmester 2016 (FUNCTION)	205	292	217	291	0.94	[0.85; 1.04]	16.4%	16.4%
Chen 2013	81	132	90	132	0.90	[0.75; 1.08]	5.2%	5.2%
Combe 2006, 2009	76	103	74	101	1.01	[0.85; 1.19]	6.1%	6.1%
Dougados 2013 (ACT–RAY)	194	276	198	277	0.98	[0.88; 1.09]	14.5%	14.5%
Edwards 2004, Strand 2006	26	40	29	40	0.90	[0.67; 1.21]	1.9%	1.9%
Emery 2009 (GO BEFORE)	82	159	98	159	0.84	[0.69; 1.02]	4.4%	4.4%
Emery 2015 (AVERT)	76	116	86	119	0.91	[0.76; 1.08]	5.6%	5.6%
Kameda 2010, 2011 (JESMR)	44	69	66	73	0.71	[0.58; 0.86]	4.5%	4.5%
Kameda 2019 (HARUKA), Sarilumab 150mg	22	30	12	15	0.92	[0.66; 1.28]	1.5%	1.5%
Kameda 2019 (HARUKA), Sarilumab 200mg	20	31	11	15	0.88	[0.59; 1.31]	1.0%	1.0%
Kaneko 2015 (SURPRISE)	77	115	86	118	0.92	[0.78; 1.09]	5.8%	5.8%
Klareskog 2004 (TEMPO)	158	223	188	231	0.87	[0.78; 0.97]	15.2%	15.2%
Kremer 2018 (COMP–ACT)	95	147	109	147	0.87	[0.75; 1.02]	7.1%	7.1%
Weinblatt 2015, Clazakizumab 100mg	35	60	39	60	0.90	[0.68; 1.19]	2.1%	2.1%
Weinblatt 2015, Clazakizumab 200mg	34	59	40	60	0.86	[0.65; 1.15]	2.1%	2.1%
**Fixed effect model**		**1955**		**1944**	**0.91**	**[0.88; 0.95]**	**100.0%**	**--**
**Random effects model**					**0.91**	**[0.88; 0.95]**	**--**	**100.0%**

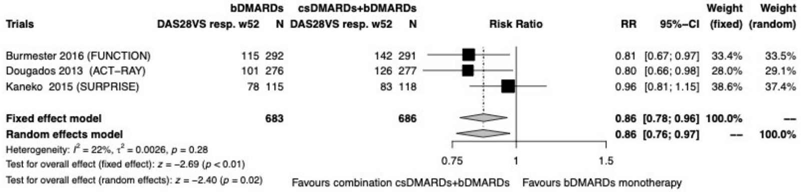

Trials	bDMARDs DAS28VS resp. w52	N	csDMARDs+bDMARDs DAS28VS resp. w52	N	RR	95%–CI	Weight (fixed)	Weight (random)
Burmester 2016 (FUNCTION)	115	292	142	291	0.81	[0.67; 0.97]	33.4%	33.5%
Dougados 2013 (ACT–RAY)	101	276	126	277	0.80	[0.66; 0.98]	28.0%	29.1%
Kaneko 2015 (SURPRISE)	78	115	83	118	0.96	[0.81; 1.15]	38.6%	37.4%
**Fixed effect model**		**683**		**686**	**0.86**	**[0.78; 0.96]**	**100.0%**	**--**
**Random effects model**					**0.86**	**[0.76; 0.97]**	**--**	**100.0%**

Copyright © 2020 Author Names and Contact Details

Figure S1. Original RCTs.
